# Underlying strategies of scaling up and sustaining physical activity interventions

**DOI:** 10.3389/frhs.2026.1767433

**Published:** 2026-07-15

**Authors:** Hatem H. Alsaqqa, Abdallah Alwawi

**Affiliations:** 1 Public Health Department and Deanship of Scientific Research, Al-Quds University, Jerusalem, Palestine; 2 Palestinian Ministry of Health, Gaza, Palestine; 3 Nursing Department, Faculty of Health Professions, Al-Quds University, Jerusalem, Palestine; 4 INTI International University, Putra Nilai, Negeri Sembilan, Malaysia

**Keywords:** intervention, large-scale, physical activity, scaling up, strategies, sustainability

## Abstract

**Background:**

Very few effective physical activity interventions are magnificently translated. However, rare interventions have successfully scaled up and sustained to achieve large-scale physical activity promotion.

**Aim:**

The study aimed to determine the contextual elements and variables that support physical activity interventions for successful scaled-up and sustained implementation.

**Methods:**

The following study employed a scoping review to evaluate the current state of physical activity interventions. The search strategy depended on three electronic databases (EMBASE, PubMed and ScienceDirect). Considering the inclusion criteria, published articles between 2015 and 2025 were included in this review.

**Results:**

Fifteen articles, eight randomized control trials, a single study of perspective, longitudinal research, in-depth interviews, quasi-mixed methods approach, intervention program, pilot assessment, and multidisciplinary workshop were included in the analysis of this study. We propose strategies for researchers to enhance the likelihood of successfully implementing, scaling up and sustaining physical activity interventions in real-world settings.

**Conclusion:**

We summarize the elements that can contribute to a higher success rate when it comes to scaling up physical activity interventions around the world.

## Introduction

Increasing physical activity (PA) can prolong life, lower mortality and the risk of chronic disease, and enhance health (1). The significance of PA for health is important for population groups with different socioeconomic status in dissimilar nations. The focus of PA research has expanded over the past half-century from an almost sole emphasis on fitness to include many choice-based areas of PA for health, such as walking for transit, household tasks, and social interaction (2).

Thus, the World Health Organization (WHO) has set a goal of a 15% relative reduction in the global prevalence of physical inactivity among adults by 2030 by implementing community-wide or whole-of-community activities. Programs that target the entire community on several levels in an effort to promote behavioral changes are known as community-wide programs (3).

As PA interventions given in controlled environments show encouraging results, interventions must be successfully scaled up to reach broader population-level health benefit. In order to do this, interventions are created with scalability in mind (4), and scale-up or implementation frameworks serve as implementation guidelines. In the end, scale-up and sustainable implementation depend on multi-levels and multi-sectors (5), cooperative partnerships, continuous stakeholder interactions (6), and a clear progression through implementation periods.

This kind of scale-up could occasionally be the initial step, in our view, however scaling up is not just accomplished by scholars spearheading the execution of a translated program at larger levels (such as at the local or state level). Successful scale-up can only be deemed accomplished when an intervention transcends the research setting and is integrated into a system, guaranteeing the preservation and sustainability of its health benefits. “Scalable interventions” in these broader areas of public policy are probably required to encourage societies to lead more active lives (7).

The research community has been urged to change its emphasis from small-scale, strictly regulated interventions to those that can be translated and disseminated (8). Study designs that are better suitable for community include pragmatic trials, hybrid effectiveness-implementation trials, and participatory research approaches (9). These methods evaluate the generalizability of intervention effects, emphasize implementation goals, and involve important stakeholders.

Despite their potential, very few of these interventions go from study to practice, and those that do provide little information about sustainability or institutionalization in day-to-day operations. Implementation is the application of techniques to embrace and integrate evidence-based health interventions and modify practice patterns in specific circumstances. Scale-up refers to replicating and extending an intervention's reach into new cities, areas, or locations (8). Scalability refers to the willingness of an intervention to be implemented in more contexts or with more participants while maintaining its efficacy (10).

Sustainability in public health is defined as the continued use or delivery of an intervention in routine practice after the withdrawal of external implementation support (10). It is typically considered to occur following the completion of program implementation and the cessation of start-up funding (4). Given that meaningful changes in population health may not become evident until at least three to ten years after implementation (11), sustainability is also strongly influenced by the setting of the intervention and the outcomes it produces (12).

Nonetheless, it has been postulated that the impacts of interventions could be reduced when implemented at scale in more realistic settings; this occurrence is referred to as a “scale-up penalty”. For instance, McCrabb and colleagues discovered that cultural adjustments were made to deliver health interventions to various population groups (13), and that culturally tailored interventions can be more effective. These difficulties stem from the social-ecological distinctions between testing a physical activity intervention in a worth pilot under idyllic circumstances and dissemination, implementation, and scale-up trials, where multilevel structure, properties, demographics of participants, and values are far more adjustable (14).

Theories, models, and frameworks do not offer guidance on how to start contextually relevant processes to engage and collaborate with typical community or healthcare groups, despite promising methodological advancements to promote diffusion, implementation, and scale-up trials (15).

A theoretical framework for creating a cohesive approach to organizing interventions to enhance PA is provided by the social ecology model of behavior change, which considers several levels of impacts on people's behavior. Essentially, our goal is to discover actions to reintegrate active living into the areas of government and society where it once existed—urban planning, transportation, education, culture, leisure, environmental sustainability, and health—by looking for efficient ways to scale up physical activity interventions globally.

In order to promote behavioral changes, community-wide programs are interventions that are attractive to the overall community on several levels (16). According to research, populations and the intricate relationships between factors should be the main focus of PA promotion initiatives. Few research studies have looked at how its factors might change depending on socioeconomic position or within populations that are economically disadvantaged (17). Although scholars have developed and (re)formulated numerous theoretical models of behavior change, lay understandings of PA behavior have received relatively little consideration within these frameworks (18).

To evaluate intervention adjustments and how they affect the scalability of an intervention, more research is required. No thorough evidence synthesis has been conducted on the effects and/or variations of scaled-up physical activity interventions in large-scale settings.

In order to add to the expanding body of evidence for implementing health promotion of PA interventions at scale, we set out this review to close this evidence gap. This review's specific goals were to: (1) describe strategies used by PA interventions during the scale-up and sustainment process; and (2) examine the PA interventions' themes used in large-scale community settings. The authors determined the following definitions to make this work more simple for reading and clear for further scientific research in the field.

### Operational definitions

1. Implementation: activities used to deliver an intervention within a specific setting (training, workflow integration, support, monitoring).
2. Scale-up: deliberate expansion of an intervention beyond the original setting or population, including horizontal expansion and vertical/policy integration.
3. Scalability: the potential or readiness of an intervention to be expanded while retaining acceptable feasibility and effectiveness.
4. Sustainability: continued delivery and maintenance of intervention activities and benefits after initial implementation support or research funding has ended.

Nevertheless, frameworks identified in the literature were categorized into two broad groups. First, behavioral or intervention-level frameworks (e.g., Social Ecology Model and other behavior-change theories) were used to inform intervention design. Second, implementation and scale-up frameworks (e.g., RE-AIM, WHO/ExpandNet, CFIR, PRACTIS, and related approaches) were used to guide the adoption, implementation, and sustainability of interventions. While both framework types were reported in the included studies, the primary focus of this review was on implementation, scale-up, and sustainability strategies rather than on the behavioral mechanisms.

## Methodology

### Design

The goal of a scoping review, which is analysis of the literature, is to quickly identify the main concepts directing a research question as well as the primary sources and kinds of evidence that are available, particularly when the topic is complicated or has not been thoroughly investigated previously (19).

This type of scoping review illustrates the body of information that already exists within the confines of the study field rather than concentrating on particular study findings (20). As shown below, data were searched and incorporated into subsequent phases. In compliance with the standards for conducting research papers, peer reviews, and systematic reviews, a scoping review was organized and carried out. Even though the articles' quality does not fall under the purview of the usual scoping review analysis, the two researchers completed a brief descriptive appraisal using the appropriate JBI critical appraisal checklist for each study design (e.g., randomized trial, qualitative study, quasi-experimental study) to provide context for interpreting the evidence. Ratings were summarized as low, moderate, or high methodological concerns and were used descriptively as they did not determine study inclusion.

In this study, we present a summary of elements that may contribute to a higher success-to-failure ratio when it comes to scaling up and maintaining physical activity initiatives globally.

The primary objective of the review was “What are the strategies and approaches being used for scale-up intervention, and sustainability being used for PA promotion?”.

Arksey and O'Malley (20) propose using a broad definitional approach and suggest that, after the complete sequences of information within a particular area has been attained, search words can be modified and summarized subsequently to handle bibliographic references. Because it uses a comparable analytical framework for all studies—a practice thought to be common in scoping reviews—this methodology demonstrates a “descriptive-analytical” approach to projecting. The articles were used to collect both qualitative and quantitative data for this investigation. This study's main objective was to conceptually characterize the methods utilized to implement large-scale PA promotion strategies.

### Search methods

An electronic search of PA interventions was conducted, and three databases were identified. The PubMed, ScienceDirect and Embase databases were searched by the authors. We searched through the abstracts and article titles for the terms “physical activity, exercise, intervention, large-scale, scale up, and sustainability.” Based on the purpose of the study, it was determined that these words should be utilized the most (Appendix 1). Duplicate articles were eliminated, and at this stage the remained number of searched articles was 51 articles.

### Inclusion criteria

**Figure 1:**
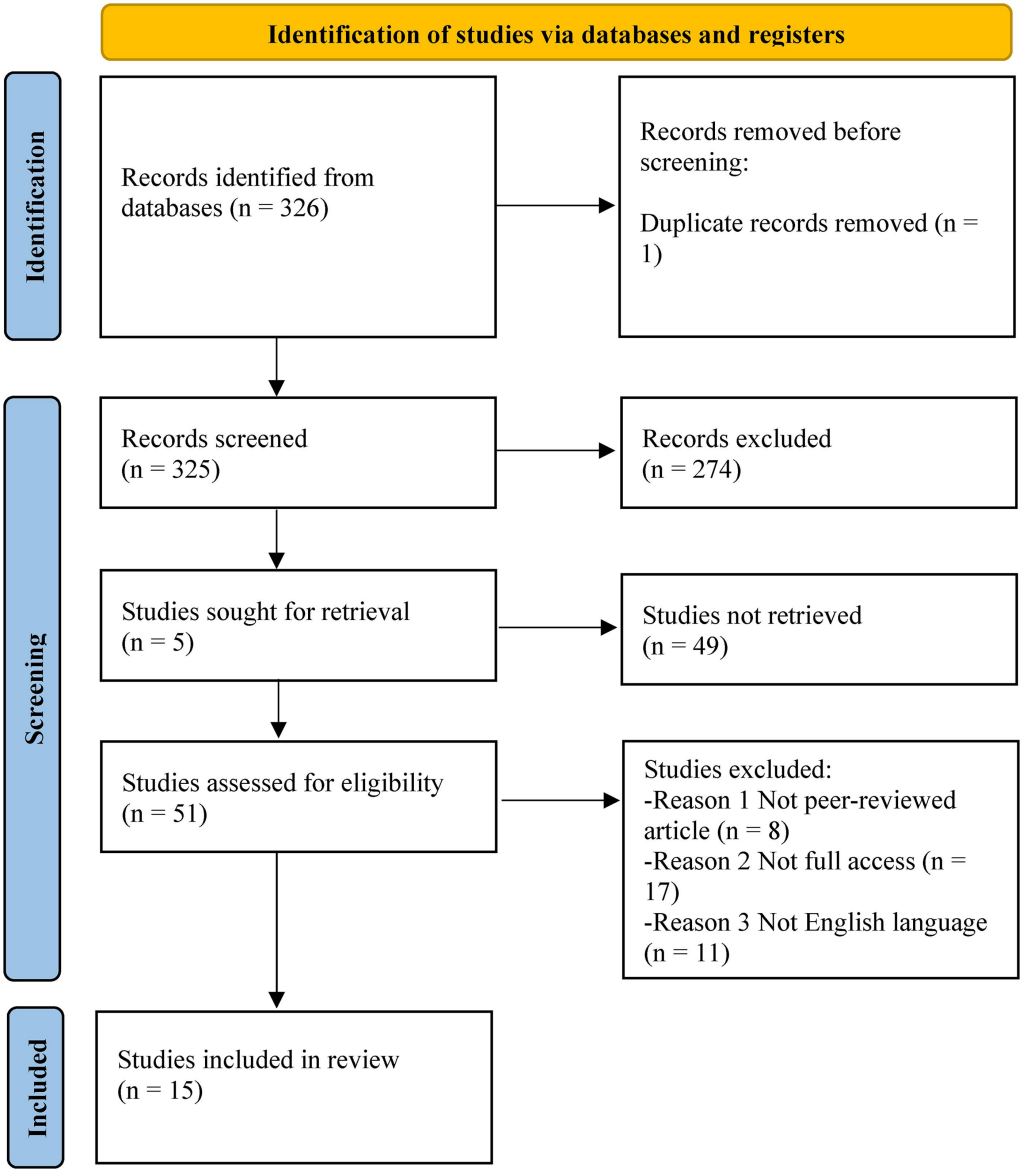
Prisma flow chart of the scoping review search.

A thorough search of the peer-reviewed literature was one of the four main eligibility requirements for academic research (Figure 1):

- Research or peer-reviewed publications that have already been published;
- Full access papers;
- Written in English studies
- Articles released between January 1, 2015, and October 1, 2025

The total number of the eligible articles as this phase reached 15 articles.

### Exclusion criteria

Studies were excluded if they did not fit the previously indicated criteria. Studies on clinical program management were included, however studies on protocols, systematic and scoping reviews were excluded.

### Data extraction

A. H. H. and A. A. analyzed and critically assessed the studies. Each research study's key findings were extracted, and a three-stage literature screening procedure that comprises evaluating the complete text, abstract, and title of each study was followed. However, the two authors independently screened the records twice and subsequently reached consensus through discussion.

Differences at screening levels were examined and updated.

Stage 1: The following details were gathered from each study that was taken into consideration (Table 1): author, setting and design, aim, key findings, and recommendations

**Table 1:** A summary of reviewed studies.

Author	Setting/design	Aim	Main findings	Recommendations
Haughton et al. (21)	In-depth interviews USA	To encourage Latina women who attend church to engage in moderate-to-intense physical activity.	Faith-based health promotion programs can be scaled up through four promising implementation strategies: (1) training pastors and staff on health behavior change; (2) customized messaging; (3) community collaborations; and (4) denominational support.	- It is necessary to identify and comprehend certain implementation obstacles and enablers. - Improving program implementation and paving the road for scale-up and diffusion can be achieved by addressing these obstacles at the adopter and organizational levels through focused implementation methods.
Hoekstra et al. (22)	Longitudinal data Netherlands	To shed light on how variations in the fidelity trajectories of health promotion program's delivery in multiple disciplines relate to advances in patients’ health behavior.	- The conditions of the local organization determine the trajectory of effective implementation fidelity. - Maintaining the program's uniform straightforward components, involving the limited number of physicians throughout the process, and realizing a localized change vision were all necessary to achieve stable high implementation fidelity.	It suggests that while expanding evidence-based health promotion initiatives, implementation conflicts should be managed according to the initial status, size, and situation of local organizations.
Marshall et al. (23)	Discrete intervention components Australia	To take a look at changes for scaling up, it maps and contrasts the distinct intervention components of the scaled-up version and the original randomized controlled trial.	Reducing the number of sessions, expanding the range of experts leading the groups, substituting tangible instruments and hard-copy materials with a mobile app for parents, and expanding the content were some of the main changes made to the delivery model during scale-up.	- As the randomized controlled trial was expanded, it offered crucial insight into what and why the intervention's components were changed. - Analyzing these intervention changes offers crucial information on outcome impacts, the viability of scaling up, and how to best apply strategies for population-level assistances.
Fichtner et al. (24)	Randomized controlled Trial Germany	To assess the 12-week multimodal web-based PA program's efficacy.	It draws attention to the possibility of digital interventions in reducing physical inactivity, implying that the quality and applicability of the information offered may be just as important to these programs’ success as their interactivity.	Optimization measures for such treatments, particularly for people with low PA, such as user engagement, behavior modification strategies, and the incorporation of objective PA tracking approaches, require more investigation.
Maher et al. (25)	Randomized Controlled Trial Australia	To assess an online social networking physical exercise intervention's viability, effectiveness, and engagement.	- Self-reported weekly moderate-to-vigorous physical activity (MVPA) served as the main outcome measure. - Weekly walking, time spent engaging in vigorous and moderate physical activity, overall quality of life, and mental health quality of life were secondary outcomes. - Random-effects mixed modeling was used for the analyses, taking into consideration possible team-level clustering. - In order to assess feasibility and engagement, usage statistics were presented in a descriptive manner.	Significant short-term increases in physical activity can be achieved by an online social networking physical activity intervention using pedometers.
Scheffey et al. (26)	STEP together trial USA	To test wearable technology combined with social incentives to promote physical activity among families in the Philadelphia area, the study's design and baseline participant characteristics are described.	- Interventions that combine wearable technology with social incentive techniques based on behavioral science may be scalable, long-lasting, and successful. - A sizable community-based experiment called STEP Together is evaluating gamification and charitable contributions as tactics to encourage families in the Philadelphia region to walk more each day.	- It adds to the increasing amount of data regarding scalable, successful methods for encouraging people and families to involve in more physical exercise on a daily basis.
Hasson et al. (27)	A quasi-mixed methods approach USA	To: (1) provide techniques for methodically identifying implementation impediments at the organization and system levels; and (2) connect organizational priorities and system policies through the construction of multi-level implementation strategies.	Three implementation obstacles were identified: a lack of structural capacity to support teacher training, a lack of resources across districts and school buildings to assist teachers, and a misalignment of the policies and priorities of the district PA and the intermediate school district.	To ensure that PA interventions are implemented and sustained effectively, more focus should be placed on organizational capability and the infrastructure that is already in place.
Lyons et al. (28)	A Randomized Controlled Pilot Trial USA	To assess an intervention's viability, acceptability, and impact on physical activity.	The population found the intervention to be both practicable and acceptable. The impact sizes were comparable to those observed with other wearable electronic activity monitors, suggesting that wearable activity monitors may be a useful tool for promoting physical activity and reducing sedentary behavior when paired with telephone counseling.	These systems exhibit potential as scalable, reasonably priced ways to deliver evidence-based behavior modification strategies. More research is required to fully comprehend how and why monitoring strategies could promote physical activity.
Hicks et al. (29)	Perspective article USA	To offer interdisciplinary advice on how mobile technology should be developed and used in order to encourage actions that benefit general health.	In addition to assessing the connections between mobile technology data and health in order to develop evidence-based procedures, it advances the personalization of interventions to an individual and their social, cultural, and contexts.	It advances the application of human-centered, tailored, and theory-based strategies for encouraging healthy behaviors.
Pynnönenet al. (30)	A two-arm single-blinded randomized control trial Finland.	To investigate if modifications to theory-based constructs could be responsible for a shift in activity level through secondary analysis	The variation in activity frequency was directly described by baseline attitude and attitude changes in the improved integrated model. Indirect routes that were statistically significant were found between baseline autonomous motivation and baseline attitude, as well as between activity frequency and changes in attitude.	Significant change paths were identified by the improved integrated model, which emphasized autonomous motivation and attitudes as important change constructs.
Nettlefold et al. (31)	A cluster randomized controlled trial Canada	To (1) outline strategies that facilitated implementation and scale-up; (2) assess implementation [the delivery of physical activity (PA) by teachers] and students’ PA and CRF; and (3) examine the connections between implementation at the teacher level and student-level findings.	With continued assistance from the government and school-community stakeholders, whole-of-school PA interventions can be scaled up and maintained over a longer period of time (>10 years). Implementation resource teams must come up with innovative strategies to maintain the advantages of expanded school-based health-promoting activities in order to enhance population-level student health, despite several obstacles. A key component of scale-up success is support units with existing relationships to schools, which are made up of researchers, government stakeholders, and practitioners from the school community.	To improve student health at the population level, we need to think of innovative approaches to sustain scaled-up health-promoting programs.
Tan et al. (32)	Structured FPT program Singapore	To examine the viability and possible impact of a 12-week organized Functional Power Training (FPT) program for older individuals with high velocities and low loads.	It indicates that completing an organized 12-week high-volume, moderate-intensity FPT exercise program is both possible and safe for frail and prefrail older individuals living in the community. A larger multisite randomized control study could be conducted to assess the program's efficacy in conjunction with a health promotion social enterprise and a site-based community service provider.	For frail elderly people, community-based structured FPT is safe, practical, and has the potential to enhance function and reverse frailty status.
Wong et al. (33)	Pilot evaluation Hong Kong	To examine whether children's psychosocial wellbeing, level of physical activity (PA), and health-related quality of life (HRQOL) were improved by the Family Move app-based intervention.	The study offers initial proof that a mobile app-based intervention that incorporates basic parent-child activity techniques with gaming components may encourage PA and lessen children's psychological issues. It can be joyful and enjoyable to exercise with parents and kids using a points and level system, which may further improve the likelihood that the intervention will be successful. In order to disentangle the intervention mechanism, this study also demonstrates how to evaluate participants’ level of adherence to the intervention using points.	Through parent-child activity, the Family Move app may be a potential intervention to raise children's PA level and psychosocial wellness.
Koorts et al. (15)	Multidisciplinary two-day workshop Australia	To investigate the obstacles and enablers decision-makers encounter while scaling interventions that are unrelated to official scale-up research trials.	It addresses some of the paradoxes (things that go against expectations) and scale-up tensions (difficulties and conflicts) that emerged from this workshop in light of the most current research and experiences in this part. Scale-up conflicts are framed in terms of partnerships, time, methodology, and epistemology; paradoxes are described as “reach without scale,” “planned serendipity,” and “simple complexity.”	It proposes strategies for promoting physical activity in the future and advances knowledge of the dynamics of research-practice partnerships.
Leeman et al. (34)	Multiphase approach USA	To explain how stakeholders were involved in developing plans to expand the Med-South Lifestyle Program through a multi-phase process	Among other factors, inadequate staffing, conflicting consequences, and protection anxieties during COVID-19 were determinants of scale-up. Tailoring produced two tiers of strategies for execution. At the delivery system level, strategies included implementation teams, plan, and periodic minor testing of change. Technical assistance, training, instructional materials, and quality monitoring were all part of the support system level strategies. With conflicting results on faithfulness, it offers proof of the implementation strategies’ feasibility, tolerability, and reach.	With an emphasis on adapting two-level implementation strategies, it demonstrates how a multiphase approach was employed to get ready for the statewide scale-up of a health intervention. Planning for the scale-up of interventions athwart various delivery and care systems could be done using this method.

Stage 2: The researcher created the data-gathering worksheet used for the full-text review. The analysis process involved identifying the problem (whether it was scaling up or sustainability), searching the literature, and presenting ideas, concepts, and themes.

Stage 3: Following the authors' extensive review of the articles, a mind map was made to highlight specific ideas that were mentioned in the involved articles. This map illustrates and charts the body of knowledge within the parameters of the research field rather than going into specifics about study results (20). Since conceptual comparations and modifications served as a foundation for deciding on the final stage themes, this approach aids the authors in defining the themes of the most important research questions. Any duplication was removed after the themes (strategies, concepts, and approaches) were clearly delineated and categorized, or if they existed, removed.

### Quality appraisal

Methodological quality was assessed using the appropriate Joanna Briggs Institute (JBI) Critical Appraisal Checklists according to the study design. The included studies demonstrated generally moderate to high methodological quality. Most studies clearly stated their objectives, employed appropriate methodologies, and reported findings transparently. Randomized controlled trials generally showed adequate intervention descriptions and outcome measurements, while observational and qualitative studies provided clear research aims and contextual information. Common limitations included insufficient reporting of recruitment procedures, limited discussion of confounding factors, incomplete descriptions of implementation fidelity, and inadequate reporting of long-term sustainability outcomes. As recommended for scoping reviews, no studies were excluded based on quality appraisal; rather, the assessment was used to provide context for interpreting the evidence.

The authors developed a table to present a structured overview of the included studies, their designs, the appropriate JBI appraisal tools, and a descriptive summary of methodological quality (Table 2).

**Table 2:** Joanna Briggs institute (JBI) critical appraisal summary of the included studies (*n* = 15).

Study	Design	JBI checklist used	Overall appraisal	Main methodological strengths	Main methodological concerns
Haughton et al. (21)	Qualitative (In-depth interviews)	JBI Qualitative	Moderate	Clear objectives; appropriate methodology	Limited reflexivity reporting
Hoekstra et al. (22)	Longitudinal study	JBI Cohort	Moderate–High	Longitudinal follow-up; clear outcomes	Confounding factors incompletely addressed
Marshall et al. (23)	Intervention scale-up analysis	JBI Cross-sectional	Moderate	Detailed adaptation description	No comparison group
Fichtner et al. (24)	RCT	JBI RCT	High	Randomization; valid outcomes	Limited long-term maintenance reporting
Maher et al. (25)	RCT	JBI RCT	High	Robust analysis	Attrition and sustainability not fully explored
Scheffey et al. (26)	Implementation study	JBI Quasi-experimental	Moderate	Strong implementation focus	Effectiveness outcomes limited
Hasson et al. (27)	Quasi-mixed methods	JBI Mixed Methods	Moderate	Multi-level implementation analysis	Limited causal inference
Lyons et al. (28)	Randomized Pilot Trial	JBI RCT	Moderate–High	Feasibility well reported	Small sample size
Hicks et al. (29)	Perspective article	Not formally appraisable	Contextual evidence	Implementation insights	Opinion-based
Pynnönen et al. (30)	RCT	JBI RCT	High	Strong theoretical framework	Sustainability not assessed
Nettlefold et al. (31)	Cluster RCT	JBI RCT	High	Large-scale assessment	Fidelity variation
Tan et al. (32)	Feasibility study	JBI Quasi-experimental	Moderate	Feasibility and safety evaluated	No control group
Wong et al. (33)	Pilot evaluation	JBI Quasi-experimental	Moderate	Adherence measured	Small sample size
Koorts et al. (15)	Workshop	Not formally appraisable	Contextual evidence	Scale-up insights	Expert opinion
Leeman et al. (34)	Multi-phase implementation study	JBI Quasi-experimental	Moderate–High	Comprehensive planning	Limited effectiveness evaluation

## Results

The investigation of this analysis included fifteen publications; 8 randomized control studies and one study of each; perspective, longitudinal study, in-depth interviews, quasi-mixed methods approach, intervention program, pilot evaluation and multidisciplinary workshop study. Six of the investigations were supported out in the United States, three in Australia, and one each in Germany, Canada, the Netherlands, Finland, Singapore, and Hong Kong.

The authors investigated the reviewed articles about the most relevant methods and strategies for scaling up and sustaining PA interventions (for large-scale settings) in various populations for further information. Moreover, mutual strategies that can be used by other health promotion interventions but not specified for PA were not further discovered since they fell outside the purview of this investigation. Most randomized and quasi-experimental studies were judged to have low-to-moderate methodological concerns, whereas perspective/workshop papers were treated as contextual evidence rather than effectiveness evidence.

Moreover, the included studies were heterogeneous in design, population, setting, and country. Therefore, the authors did not attempt quantitative assembling or compare effect sizes across studies. Instead, we conducted a thematic synthesis focused on implementation, scale-up, and sustainability processes. Studies were grouped by their primary contribution to one of three domains: (1) implementation strategies, (2) scale-up approaches, and (3) sustainability factors

The following strategies and methods were identified as describing the process of increasing PA programs and initiatives: engaging stakeholders in the process (23, 34) vertical scale-up and horizontal scale-up (21, 23, 32, 34) socio-economic status and system approaches (15, 25, 30) multidisciplinary PA interventions and cultural barriers (23, 30) determinants to program implementation (34) technology and scalable measurements (24, 26, 28, 29, 33) and sustainability strategies (21, 22, 27, 31) (Appendix 2).

### Engaging stakeholders in the process of research translation

Our findings confirm recent recommendations to involve target communities early in PA programs and offer instances of the benefits of resident involvement (35). Additionally, the findings demonstrate how public participation, which identifies local issues and effectively sheds light on community attitudes, could influence both research advance (36) and the design of interventions targeting disparate populations. Recent systematic reviews have consistently shown that community engagement, self-efficacy, and social support are important determinants of physical activity participation and intervention sustainability. Reviews by Shoesmith et al. (37), Bodkin and Hakimi (38), indicate that collaborative partnerships and stakeholder involvement enhance intervention adoption, implementation, and long-term maintenance. These findings support the importance of actively involving communities throughout intervention planning and implementation.

The knowledge of ordinary citizens and community leaders should be shared with those who develop PA interventions in order to maximize opportunities, guarantee safety, and provide suitable facilities for everyone. In order to give residents PA advice, leaders recognized the need for a qualified community resources. Raising locals' understanding of the importance of PA for health may encourage cooperation among community organizations and help them succeed in their funding applications (39).

### Two directions of scale-up PA interventions

Scaling up public health initiatives at the niche level implies a shift into standard practice at the management level (i.e., integration into the present health system). This shift is influenced by the environment [i.e., contextual factors such the prevailing norms and values (34)]. The structure (organizational method), practice (way of doing), and culture (method of thinking) must all be altered or reconfigured in order to move from a specialty practice to an universal practice at the regime level (39).

Horizontal scale-up and vertical scale-up are different strategies. Horizontal scale-up is the expansion or duplication of an intervention, but vertical scale-up relates “to the policy, legislative, regulation, economic or other health systems changes required to strengthen the innovation at the national or sub-national level” (40). Vertical scale-up is essential to ensuring that scale-up is sustainable and that the enhanced modifications will persist after the scale-up process has concluded (40).

The “multi-level perspective on transition” is one paradigm for comprehending transitions. This paradigm depicts the collaboration of processes at three analytical levels: niches, regimes, and landscape (40). It sees transitions as non-linear processes. According to Van den Bosch and Rotmans (39), a niche is “a specific type of societal subsystem,” which could be thought of as the pilot intervention in the case of scale-up. The regime comprises “the dominant structure, culture, and practices through which actors interact.” The landscape comprises “the broader societal trends and contexts of transitions, such as demographics, culture, and values” (41).

### Multidisciplinary aspects of PA interventions

A variety of factors influence individual capacity, opportunity, and incentive to modify their PA. Giving communities a sense of ownership should make people feel more empowered and capable. To enable strategic planning and prevent needless copying of labor and properties, multilevel interventions (42) and multidisciplinary teamwork are required. Additionally, PA health promotion messages must be revised in both content and delivery to ensure that underprivileged groups understand their personal significance. In order to boost confidence and beliefs, PA information should be “clear and consistent” when given to these groups (43).

The scaling up of physical activity programs can be better understood and enhanced by applying theory, rationale models, and systematic planning approaches (44). A planning framework's primary goal is to map the important connections, phases, and circumstances that could influence scaling up and from which conclusions on efficacy could be drawn. A framework can assist in making sure that academics concentrate on the most important aspects of the scaling-up process and that practitioners and policymakers are aware of its tiered character (e.g., that sustainability requires efficacy) (8).

### Determinants to intervention implementation

According to research, populations and the intricate relationships between factors that contribute to physical inactivity should be the main focus of PA promotion interventions (45). Although, several factors influence physical inactivity, individual-level research has been the primary focus of studies on its implementation determinants. How its determinants might differ with socioeconomic status or within socioeconomically disadvantaged populations has not been extensively explored.

However, a theoretical framework for creating an integrated approach to organizing interventions to enhance PA is provided by the social ecology model of behavior change, which considers several levels of impacts on people's behavior. It acknowledges the significance of individual's ideas and abilities as well as their physical surroundings and the social and cultural standards of their group (46).

### The impact of the socio-economic status

Fewer studies have tested the application of current theories and causal mechanisms across several levels and identified elements that either support or undermine the scaling up and sustainability of PA interventions from a complex system level (47).

Four important criteria that stakeholders believed influenced the sustainability of PA interventions were identified. These elements reflect the difficulties in putting into practice a strict and uniform “one-size-fits-all” program across many levels, demographic locations and structures, as well as conflicting policies and sociopolitical disorder.

However, two challenges were associated with the changing political and economic environments: competing interests and finance, as well as political cycles. It is increasingly recognized that “new factors” influencing sustainability include short budget cycles, internal political restrictions, and governmental changes (47). Although these factors are rarely taken into account, population delivery is especially susceptible to changes in the social, economic, and political environments.

These findings are especially pertinent as the literature grows to examine factors related to sustaining policies (36, 47). Conflicting stakeholder interests, variations in local, regional, and state policies, and their integration are starting to be acknowledged as significant variables in determining whole-of-system initiatives in implementation.

Nonetheless, complex health challenges that cannot be resolved by straightforward programs and uniform implementation are being addressed through a deeper knowledge of systems approaches to health promotion (48, 49). A whole-of-system approach is also required for the enablement of tangible implementation of scaling up PA interventions to sustainability.

### Technology and scalable measurements

In today's world, smartphones and reasonably priced wearable sensors are commonplace. The creation and implementation of remote PA interventions may benefit from these devices. The capacity to plan the delivery of intervention content that can account for the user's current environment and time of day is one of the benefits of smartphones and devices incorporated into smartphone platforms.

As a result, smartphone-based interventions can provide low-touch or fully remote interventions and are easily accessible, scalable, and reasonably priced. These features make smartphone-based interventions superior to computer-based interventions and more beneficial for self-monitoring PA when compared to standalone pedometers (50).

Moreover, several studies included in this review demonstrated the acceptability, usefulness, and efficacy of smartphone-based interventions in promoting healthy behaviors across a range of age groups. Furthermore, mobile technology has the ability to reveal the causes and consequences of health behaviors by using high-resolution, real-world data from an increasing number of available sensors (29). Fichtner (24) et al. described positive outcomes from a web-based intervention, while Wong et al. (33) demonstrated the feasibility of a family-based mobile application to improve physical activity among children. Similarly, wearable technology interventions evaluated by Lyons et al. (28) and Scheffey et al. (26) showed potential for scalable and sustainable delivery of behavior-change strategies.

### Sustainability strategies for physical activity interventions

Across the included studies, several strategies were identified as important for sustaining physical activity interventions beyond the initial implementation phase. First, integration of interventions into existing organizational and community structures was consistently associated with long-term maintenance. Programs embedded within schools, community organizations, faith-based settings, and healthcare systems were more likely to continue after external support was withdrawn (21, 22, 31). Second, stakeholder engagement and leadership support emerged as critical sustainability factors. Ongoing collaboration among researchers, policymakers, practitioners, community leaders, and participants facilitated continued program delivery and adaptation to local needs (15, 21, 31, 34).

Third, workforce development through training, technical assistance, and implementation support strengthened organizational capacity to sustain interventions over time (21, 27, 34). Additional sustainability strategies included policy alignment and vertical scale-up, which enabled interventions to become incorporated into routine practice and broader health systems (15, 27, 40). Continuous monitoring, quality improvement activities, and implementation support teams also contributed to maintaining intervention fidelity while allowing necessary contextual adaptations (22, 31, 34). Finally, community ownership, organizational commitment, and stable funding mechanisms were repeatedly identified as essential factors for ensuring long-term sustainability and maximizing population-level impact (6, 12, 36, 47, 51).

Overall, the included studies demonstrated that successful scale-up and sustainability of physical activity interventions depend on a combination of intervention, organizational, and system-level factors. The most frequently reported strategies included stakeholder and community engagement, leadership support, organizational readiness, policy alignment, workforce training, and the use of digital technologies to enhance reach and accessibility. Sustainability was promoted through integration into existing systems, long-term stakeholder involvement, continuous monitoring and evaluation, and institutional support. Across studies, interventions that combined community participation with supportive organizational and policy environments appeared to have the greatest potential for achieving both scale-up and long-term sustainability.

## Discussion

This scoping review aimed to explore the strategies and methods utilized for the scalability and sustainability of large-scale PA interventions. Our results support previous recommendations to involve target communities from the beginning of PA programs and offer examples of the benefits of incorporating citizens.

Additionally, the studies' findings demonstrate how public participation, which identifies local issues and sheds light on community attitudes, could influence research development (52) and the design of interventions specifically targeting underprivileged populations (35). However, residents' readiness to participate in current interventions was negatively impacted by their unsatisfactory experiences with prior initiatives; therefore, actively involving them in planning and delivery should simplify communication with service providers and backing their long-term involvement in such interventions.

The first step in tackling these variables during the scale-up process is to design a scale-up strategy before scaling up. Focusing on how to manage the scale-up process by facilitating changes in arrangement, practice, and culture within the current site is crucial to this scale-up strategy. WHO/ExpandNet (40) created a practical guideline paper that outlines nine phases for creating a scale-up strategy. The main strategic decisions they concentrate on are the invention itself, the user organization's capability, the environment, and the vertical and horizontal scale-up.

Moreover, the interdependence of stakeholder engagement, leadership support, organizational capacity, workforce readiness, funding availability, policy alignment, intervention adaptability, monitoring systems, and community participation highlights the importance of adopting a comprehensive scale-up strategy in which all pertinent stakeholders are meaningfully involved and all factors influencing scale-up are taken into consideration. This is consistent with WHO/ExpandNet's scale-up perspectives, which explain that institutionalization and growth occur within a complex network of relationships. Thus, one of the fundamental ideas that directs scale-up is systems thinking (53).

The sustainability of the scale-up is strongly assumed to require systemic adjustments and institutionalization, which is also emphasized in a number of other publications and advice materials (WHO/ExpandNet) (40, 54). Sustainability is jeopardized when scale-up just concentrates on the horizontal extension of an intervention. An emphasis on vertical scale-up is necessary for Universal Health Coverage because sustainable scale-up of PA initiatives is essential; this thinking can be guided by the multi-level viewpoint (55).

But the structure that effectively captured the essential components was the RE-AIM framework. Reach, Efficacy/Effectiveness, Adoption, Implementation, and Maintenance are all measured in stages by RE-AIM (56). The term “reach” in RE-AIM describes the percentage of the target group that participates as well as the features of participants in comparison to non-participants; in other words, does the scaled-up intervention reach the people who are most at risk for inactivity? At the system level, adoption refers to the proportion and representativeness of organizations that will embrace a particular program or policy. For example, how many states, municipalities, and school districts in a country, as well as a municipality, adopted a school-based intervention as an official school program to be employed within their influence?

Furthermore, RE-AIM has been used in a wide range of situations, diseases, and risk factors. It has also been shown to be helpful in assessing the results of physical activity scale-up initiatives and public health strategies (55, 56). Therefore, this paradigm can be utilized to optimize the scaling up process in various stages across the research-to-practice or practice-to-research spectra by both those in the “real world” (which includes public health practitioners, stakeholders, and policymakers) and those in the “research world.” In a similar vein, researchers might use this framework to assess novel approaches that are being widely adopted without enough proof to support one or more of the RE-AIM stages (8).

Other frameworks have been used to guide implementation, scale-up, and sustainability research. These include the Consolidated Framework for Implementation Research, the PRACTIS Framework, the Normalization Process Theory, and the World Health Organization ExpandNet framework. These frameworks help the researchers understand contextual influences, implementation barriers, adaptation processes, and long-term sustainability. While several theoretical models of behavior modification have been created and updated by academics, popular interpretations of PA behavior have gotten relatively little consideration in these models.

Thus, the lack of qualitative research on lay perceptions of PA promotion is indicative of the need to better understand community linkages and interactions in order to ensure that theory is applied and to build effective interventions (57). These elements, which have to do with the individual and their environment, are crucial parts of social ecology theory. Furthermore, efficient techniques for breaking down complicated treatments to identify their “active ingredients” and handle component interactions need to be created (58).

As well, it is still difficult to apply mobile health research to clinical and public health practice. Numerous organizations, including government organizations, insurance providers, researchers and scientists, and private businesses, could work together to address this dilemma. Collaborations between these areas should provide incentives and motivation for translational work (e.g., targeted funding possibilities). This collaboration, along with other suggestions, is discussed by Tomlinson and colleagues (59) as being crucial for expanding mobile health technologies.

Translation would also benefit from further efforts to build upon current research platforms for mobile health solutions. By improving the interoperability of various data formats and removing the requirement for researchers to start from scratch with every new study, increasing these venues for multi-modal mobile health research would expedite research as well as translation (29). Instead of focusing only on the individual, research should also examine PA interventions at the population level.

While we support providing each person with tailored reaction and interventions to motivate them to alter their behavior, mobile interventions may have an even greater effect by identifying the causes and obstacles of PA health behaviors and offering data to enhance public policy and healthcare practice. To bolster this, additional efforts are required to measure and document the health and economic advantages of mobile interventions, such as disease prevention and fewer years lost to disability, and to ascertain the equity implications of mobile interventions using economic and epidemiological models (60).

Nonetheless, iterative evaluation is best utilized to improve implementation techniques for sustained implementation, distribution, and outcome assessment once scale-up is finished. In addition to facilitating efficient information sharing, teamwork, and the application of current knowledge, stakeholder conversations should seek to raise awareness of the research, practice, and policy activities related to implementation and scale up (61). During the implementation phase, it is crucial to employ strategies for effective stakeholder communication and engagement to ensure consensus on anticipated outcomes and transparency in roles and responsibilities.

Stakeholders may embody several different organizations or different roles within a single organization, depending on the context and implementation scale. Stakeholders may represent one or more “systems” (health, education, transportation systems, etc.) reliant on the type of intervention. Thus, different strategies and resources will be needed to engage various stakeholders, depending on the type of intervention, the scale of implementation, and the number of systems included in delivery. The decision tree's results will indicate potential constraints if involvement and/or engagement are below ideal levels as well as chances to fortify or find new stakeholder relationships (15).

However, understanding the system better is the next step toward enhancing PA sustainability. System dynamics modeling is a technique for figuring out how and where to intervene to increase sustainability, for example, and where to intervene to effect change in the system (62). This approach is used to find causal linkages, such as what drives the system—for example, why it only develops short-term projects—or what causes silo thinking or inappropriate plans. To get a deeper understanding of the structures that lead to specific outcomes or interactions inside the system, further analytical techniques like social network analysis and agent-based modeling can be applied (63).

Nevertheless, from a whole-of-system viewpoint, it is also necessary to better understand enabling elements for the sustainability of PA interventions, including the involvement of agencies. Self-organization, resilience, and hierarchy are characteristics of a well-functioning system (64). In order to reduce the risk of silo actions and transfer of responsibility as a means of ensuring program sustainability, hierarchy must be supported upstream, downstream, and cross-stream by a whole-of-system perspective of the various actors and their roles in the system.

### Strengths and limitations of the study

This review has several strengths. First, it provides a comprehensive synthesis of implementation, scale-up, and sustainability strategies for physical activity interventions across diverse settings and populations. Second, the review followed PRISMA-ScR recommendations and employed a systematic search strategy across multiple databases. Third, studies from different countries and intervention contexts were included, improving the transferability of findings. Finally, the review specifically addresses scalability and sustainability considerations, which remain underreported within the physical activity literature

However, understanding the constraints of the scoping review methodology is crucial. Generally speaking, scoping reviews lack the empirical rigor of meta-analyses. The confidence in the synthesized findings is influenced by the methodological quality of the included studies. Several studies provided limited detail on implementation processes and scale-up methods, which may affect the robustness and transferability of the identified strategies. However, the methodology was appropriate for this particular study.

The study's rigor has been hampered and the risk of bias has decreased because two authors completed the entire process (including the included and the extracted articles). Moreover, in order to reduce prejudice and guarantee greater dependability and transparency in the work outcomes, the authors executed the screening phases twice. Individual screening decisions were not retained separately, thus, a formal Cohen's kappa coefficient could not be calculated retrospectively. The results of this study might have been limited by search parameters such as restricting results to English-language publications, published articles (no gray literature) and full-access option. Nonetheless, the literature's shortcomings are related to the requirement for more thorough studies on the strategies of intricate interventions in the PA domain.

## Conclusion

This scoping review found that successful scale-up and sustainability of physical activity interventions depend on a combination of intervention-, organizational-, and system-level factors. Across the included studies, the most frequently reported strategies were stakeholder and community engagement, leadership support, organizational readiness, workforce capacity building, policy alignment, intervention adaptability, and the use of digital technologies to enhance reach and accessibility.

The review further demonstrated that long-term sustainability is strengthened when interventions are integrated into existing community, educational, and healthcare systems, supported by ongoing partnerships, continuous monitoring and evaluation, and adequate organizational and financial resources. Importantly, sustainability requires not only the expansion of interventions (horizontal scale-up) but also institutionalization through policy and system-level changes (vertical scale-up).

The key point is that effective physical activity interventions are not automatically scalable or sustainable. Early planning for implementation, scale-up, and sustainability, together with strong stakeholder collaboration and supportive policies, is essential to achieve lasting population-level impact.

However, further research is required to govern the most effective method for disseminating information about the significance of PA for health and well-being and to influence behavior changes that will boost PA in communities.

## Data Availability

The original contributions presented in the study are included in the article/Supplementary Material, further inquiries can be directed to the corresponding author.

## Appendix 1 Searching terms.

**Table:**

Area of databases research	Terms
Title & abstract	Physical activity **OR** exercise **AND** “intervention” **OR** “program” **OR** “initiative”
Title & abstract	**AND** Scale up **OR** scaling up
Title & abstract	**AND** Sustain **OR** Sustaining
Databases	**EMBASE**, **PubMed**, **ScienceDirect**
Types of articles	**Original articles** only
Period of research	From **2015-**till now

## Appendix 2 Refined study-to-domain correspondence for the included studies.

**Table:**

Study	Stakeholder engagement	Vertical/horizontal scale-up	Socio-economic & system approaches	Multidisci-plinary/cultural barriers	Determinants to program implementation	Technology & scalable measurement	Sustainability strategies
(15)			✓				
(21)		✓					✓
(22)							✓
(23)	✓	✓		✓			
(24)						✓	
(25)			✓				
(26)						✓	
(27)							✓
(28)						✓	
(29)						✓	
(30)			✓	✓			
(31)							✓
(32)		✓					
(33)						✓	
(34)	✓	✓			✓		
